# Genome editing: the end of the beginning

**DOI:** 10.1186/s13059-015-0860-5

**Published:** 2015-12-23

**Authors:** Jennifer A. Doudna, Charles A. Gersbach

**Affiliations:** Department of Chemistry, University of California, Berkeley, California 94720 USA; Department of Molecular and Cell Biology, University of California, Berkeley, California 94720 USA; Howard Hughes Medical Institute, University of California, Berkeley, California 94720 USA; Innovative Genomics Initiative, University of California, Berkeley, California 94720 USA; Physical Biosciences Division, Lawrence Berkeley National Laboratory, Berkeley, California 94720 USA; Department of Biomedical Engineering, Duke University, Durham, North Carolina 27708 USA; Center for Genomic and Computational Biology, Duke University, Durham, North Carolina 27708 USA; Department of Orthopaedic Surgery, Duke University Medical Center, Durham, North Carolina 27710 USA

## Editorial

It has recently become commonplace to editorialize on the extent to which genome editing has transformed modern biological research and perhaps, in the future, biomedicine. Nonetheless, each time the scope of scientific progress and the state of the field is appraised, it is followed in rapid succession by another wave of seemingly momentous developments. Each of these rounds of advances pushes at the boundaries of what can be done to the DNA within cells and organisms, expands the number of systems that can be used to engineer genomes, and increases the resolution of our understanding of how these systems work. This is also true for the articles included in this special issue of *Genome Biology* that is focused on genome editing, which provide important new insights in each of these areas. The clear takeaway from this collection is that the recent flurry of progress and excitement around genome editing marks only the beginning. Even greater challenges lie ahead for refining and applying these new technologies, interpreting their results, and deciding in what capacity they should be used.

The most sophisticated biotechnologies continue to emerge from the seemingly most simplistic organisms. Similar to the repurposing of green fluorescent protein from jellyfish for imaging studies or small interfering RNAs from worms for gene knockdown applications, the adoption of the CRISPR/Cas9 system from prokaryotes has enabled genome editing for the broad scientific community [1]. Despite the massive impact that the *Streptococcus pyogenes* CRISPR/Cas9 system has had directly on genome editing, it has also shone a light into the depths of alternative prokaryotic gene editing systems that can be mined for unique and orthogonal properties [2]. Consequently, several alternative Cas9 systems from other species have been described [3–5], and now other types of CRISPR systems that are independent of Cas9 are being engineered for genome editing applications [6, 7]. The translation of the oncoming flood of information about CRISPR-Cas systems is going to depend on technologies for deciphering the unique properties of each system, including target sequence requirements and specificity [8].

In the wake of the identification of these gene editing systems, it is critical to better understand their fundamental mechanisms of action. This will enable the extension of these tools to more diverse applications and their optimization for user-defined specifications. The expanse of mechanistic information that is currently lacking is both wide and deep. Many knowledge gaps need to be filled for each system and comparisons of these properties across systems will facilitate the construction of general rules. We are only now learning how these molecular machines find and interact with their DNA target sites [9, 10]. Similarly, the various cellular DNA repair processes that control genome editing outcomes can be harnessed efficiently and precisely only if we fully understand the mechanisms by which DNA breaks are recognized, processed, and restored [11]. A more complete understanding of the properties of these systems will similarly advance our capacity to design optimal tools and achieve the field’s long-term goal of developing accurate computational predictions of genome editing outcomes [12, 13].

With a variety of gene editing tools readily available and a thorough knowledge of their mechanisms of action, the diversity of possible applications across science, biotechnology, and medicine is immense. In science, the most widespread use of these tools has been for studying gene function [14, 15], but only a tiny fraction of the potential impact of this approach has been realized thus far. In biotechnology, there are many ways in which these tools could address societal challenges and improve human quality of life [16]. Most immediately, the incorporation of these technologies into agriculture can help to address the challenges of feeding a rapidly increasing world population. For example, studies in this issue [17–20] and others [21–24] have demonstrated the editing of plant genomes to confer resistance to viral infection and protection from drought. In medicine, the ability to manipulate any human gene sequence has tremendous potential to correct inherited diseases or augment cell therapies that are designed to attack cancers or regenerate diseased or damaged tissue [25]. However, there are still challenges to these strategies with regards to efficiency, delivery, and safety [26]. Many of these are the same challenges that the gene therapy field has worked to overcome for decades with significant success, but others are unique to genome editing.

While genome editing has provided scientists with unprecedented control over genomic DNA sequences, the next frontier of genome engineering is establishing similarly precise control over other properties of genome structure and function [27]. In particular, dozens of epigenetic marks have been reported and associated with various gene expression states. However, relatively little is understood about the functional roles of these marks in gene regulation. Genome editing platforms are now being used to recruit biomolecules that modulate gene regulation and modify epigenetic marks at specific chromosomal loci [28–34]. This work will elucidate the function and heritability of these marks and enable new strategies for controlling cell phenotype and perturbing regulatory functions in the genome. Moving forward, the critical challenges to epigenome editing are developing a suite of tools for manipulating any epigenetic mark and understanding the interdependent effects of environment and epigenetics on gene regulation.

The impact of recent developments in genome editing on science and biotechnology is immense. However, this incredible power over our heritable information also comes with a great responsibility to use it ethically and safely. This responsibility relates to both how we modify our own genomes and the genomes of our progeny, and the genomes of the species that inhabit our planet [35]. Thus, finding the appropriate balance between addressing crucial medical and environmental needs while respecting the limits of our knowledge will be a critical challenge for this whole field to consider [36].

The transformation of genome editing technologies in the last few years has also taught us a tremendous amount about scientific progress. Arguably, the rapid pace of this field has been driven as much by the scientists who have quickly shared results, protocols, and reagents with the community, as it has been driven by the facile nature of the new technologies [37]. Sharing pre-publication results, building websites to provide protocols and design algorithms, and establishing non-profit repositories to distribute key research reagents are all relatively new concepts to science that have evolved alongside genome editing technologies. There is much to be learned about how these new strategies for making research results available and transparent could similarly accelerate other fields.

In summary, the immediate grand challenges and opportunities for this field are defining the scope of possible genome editing tools, determining how they work, testing how they can be applied, and learning from our past to proceed wisely and cautiously with these new and powerful technologies. Each of these challenges discussed here is covered by the variety of content in this special issue of *Genome Biology*. Thus we hope this issue will provide a perspective for the field as it ventures out of this beginning phase of the genome editing revolution.
